# GD2 Identifies Cancer Stemness in Glioblastoma and Phytoalexin Library Screen Identifies Potential Novel Natural Inhibitors

**DOI:** 10.3390/ijms27083490

**Published:** 2026-04-14

**Authors:** Khoa Nguyen, Emily McConnell, Minh Tran, Nathan Burow, Orielle Edwards, Thomas Cheng, Jane E. Cavanaugh, Patrick T. Flaherty, Reza Izadpanah, Bridgette M. Collins-Burow, Stephen Boue, Matthew E. Burow

**Affiliations:** 1 Department of Medicine, Section of Hematology and Oncology, Tulane University School of Medicine, Tulane Cancer Center, New Orleans, LA 70112, USA; emcconnell@tulane.edu (E.M.); mtran13@tulane.edu (M.T.); orie.a.edwards@gmail.com (O.E.); tcheng1@tulane.edu (T.C.); bcollin1@tulane.edu (B.M.C.-B.); 2 Department of Pharmacological Sciences, Division of Medicinal Chemistry, Mylan School of Pharmacy, Duquesne University, Pittsburgh, PA 15218, USA; cavanaughj@duq.edu (J.E.C.); flahertyp@duq.edu (P.T.F.); 3 Department of Surgery, Tulane University Health Science Center, New Orleans, LA 70112, USA; rizadpan@tulane.edu; 4 U.S. Department of Agriculture, Agricultural Research Service, Southern Regional Research Center, New Orleans, LA 70179, USA; steve.boue@usda.gov

**Keywords:** cancer stem cells, GD2, phytochemicals, glioblastoma multiforme

## Abstract

Glioblastoma (GBM) is the most aggressive and prevalent primary brain tumor in adults, characterized by rapid growth, diffuse infiltration, and a dismal prognosis. Despite advances in conventional therapies, the median survival remains approximately one year, emphasizing the urgent need for novel therapeutic strategies. GD2, a disialoganglioside overexpressed in several malignancies, has been implicated in tumorigenesis and metastasis and has been identified as a cancer stem cell marker. While previous reports have identified high levels of GD2 expression in gliomas compared to normal brain tissue, its role in GBM stemness remains controversial. In this study, we revisited prior findings refuting GD2′s involvement in GBM stemness by replicating key tumorigenesis experiments and further explored its impact on stemness properties such as migration and metabolic plasticity. Additionally, a phytochemical screen was used to identify natural compounds as potential inhibitors targeting GD2-mediated tumorigenesis. Our findings aim to clarify GD2′s role in GBM and provide insights into novel therapeutic interventions.

## 1. Introduction

Glioblastoma (GBM), previously referred to as glioblastoma multiforme, is the most aggressive and prevalent primary brain tumor in adults, characterized by rapid growth, diffuse infiltration, and a dismal prognosis. With an incidence rate of approximately 3.19 cases per 100,000 people annually, GBM accounts for 47.7% of all malignant brain tumors [1,2,3]. Despite advancements in surgical techniques, radiation therapy, and chemotherapy, the median survival for GBM patients is 1 year, underscoring the need for novel therapeutic strategies [4].

GD2 is a disialoganglioside whose normal expression is largely restricted to the surface membranes of normal neuronal cells. Furthermore, certain tumors have also been found to express high levels of GD2, with notable malignancies, including neuroblastoma, melanoma, and sarcoma. Furthermore, it has been demonstrated to be a cancer stem cell marker for diseases such as triple-negative breast cancer (TNBC) and has also been implicated in mechanisms that regulate tumorigenesis and metastasis [5,6,7,8].

Analysis of ganglioside expression in normal brain tissue vs. various gliomas identified 0% expression of GD2 in control gray and white matter, with positive expression observed in gliomas and peritumoral tissue [9,10]. Despite the clear distinction in the GD2 expression between normal and tumor tissues, its specific role in GBM remains unclear. This manuscript aims to establish GD2 as a marker for cancer stem cells by revisiting a previous report that refuted GD2′s role in GBM stemness and by replicating a key tumorigenesis experiment conducted by the original authors [11]. We then expand upon GD2′s role in GBM stemness by investigating its effects on cell migration potential and metabolic plasticity. Lastly, we identify potential phytochemicals that can inhibit the tumorigenic potential of GD2 in GBM by utilizing a panel of natural compounds created in collaboration with the USDA.

## 2. Results

### 2.1. GD2 Positivity Is Correlated with Improved In Vitro Glioma Tumorsphere Formation and Migration

GD2 was previously reported to be highly expressed in GBM but not in normal neural stem cells [11]. However, subsequent in vitro tumorsphere formation experiments identified no statistically significant differences in the spheroid formation between GD2− and GD2+ sorted cells after 2–3 weeks of culture, leading the authors to conclude that GD2 is not involved in GBM cancer stemness. More recently, a different study demonstrated that GD2− cells can exhibit plasticity by spontaneously expressing GD2 and generating GD2+ cells; this is a unique characteristic of cancer stem cells (CSCs) in the heterogenous tumor population [12,13,14,15]. To further investigate the potential role of GD2 in GBM stemness, we decided to replicate the tumorsphere assay and analyze the formation at earlier timepoints.

**Figure 1:**
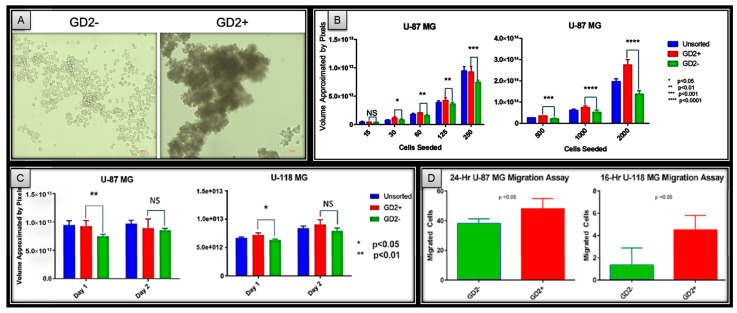
(**A**) GD2 sorted U-87 MG after 24 h in non-adherent culture conditions. Microscopy demonstrates tighter packing of GD2+ sorted cells compared to GD2−, suggesting increased tumorsphere formation capabilities. (**B**) Variable seeding of GD2 sorted U-87 MG after 24 h in non-adherent culture conditions demonstrates significantly larger sphere formation of GD2+ sorted cells compared to GD2− and unsorted controls. (**C**) GD2 sorted U-87 MG and U-118 MG spheroid sizes differences are not statistically significant after 1 day of culture, demonstrating a phenomena that will be explained in later experiments. (**D**) GD2 expression is correlated with increased transwell migratory capabilities of U-87 MG and U-118 MG cells, suggesting a positive correlation between GD2 expression and cellular motility.

Live U-87 MG cells were sorted by flow cytometry into GD2− and GD2+ groups and initially cultured in non-adherent conditions (Supplementary Figures S1 and S2). Visualization by microscopy after 24 h of culture shows more densely packed cell masses in GD2+ sorted cells compared to loose and individually distinguishable cells in GD2− sorted controls (Figure 1A). Further experiments in a 96-well plate format one passage later, with variable seeding of the sorted cells, showed that GD2+ cells exhibited significantly more robust sphere formation compared to GD2− controls, with the statistical significance increasing alongside the number of cells seeded (Figure 1B and Supplementary Figure S3). However, continued observation of sphere formation revealed a loss of differences in spheroid formation after 48 h in culture. Similar results were also observed in a second GBM cell line, U-118 MG (Figure 1C).

To investigate the migratory capabilities of GD2-expressing cells, glioblastoma cell lines U-87 MG and U-118 MG were sorted and seeded into migration assay plates containing unsupplemented media and 10% serum media separated by a transwell membrane with 3 µm pores. After incubation overnight, the transwell membrane was removed and analyzed by microscopy to determine the number of cells that managed to successfully migrate from the unsupplemented media to the 10% serum side. The results demonstrate an increased number of GD2+ compared to GD2− sorted migrated cells, suggesting that GD2 positivity is correlated with increased cell mobility (Figure 1D).

### 2.2. GD2-Expressing Glioma Cells Have Increased In Vivo Tumorigenesis Potential

**Figure 2:**
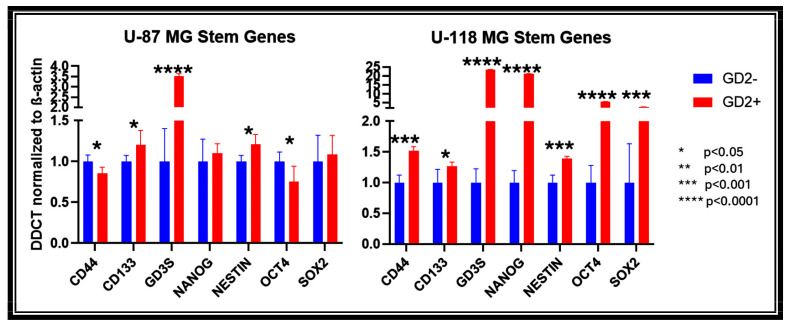
qPCR analysis of cancer stem cell genes in GD2 sorted U-87 MG and U-118 MG cells quantified by delta delta CT calculations normalized to β-actin as the housekeeping gene. Among the stemness genes studied, CD133 and nestin are positively correlated with GD3S, the GD2 rate limiting enzyme, expression in both cell lines. Other positively correlated genes include CD44, nanog, oct4, and sox2.

Based on initial in vitro data, GD2 positivity is correlated with stem-like phenotypes. To further evaluate the correlation between GD2 expression and stem-like features, qPCR analyses of GD3S, a GD2 rate-limiting enzyme, and stem cell markers (CD44, CD133, nanog, nestin, Oct4, and SOX2) were performed for sorted U-87 MG and U-118 MG cells. GD2+ sorted U-87 MG (CD133, GD3S, Nestin) and U-118 MG (CD44, CD133, GD3S, nanog, Nestin, Oct4, SOX2 cells showed a statistically significant upregulation of stem genes compared to their GD2− sorted counterparts (Figure 2).

**Figure 3:**
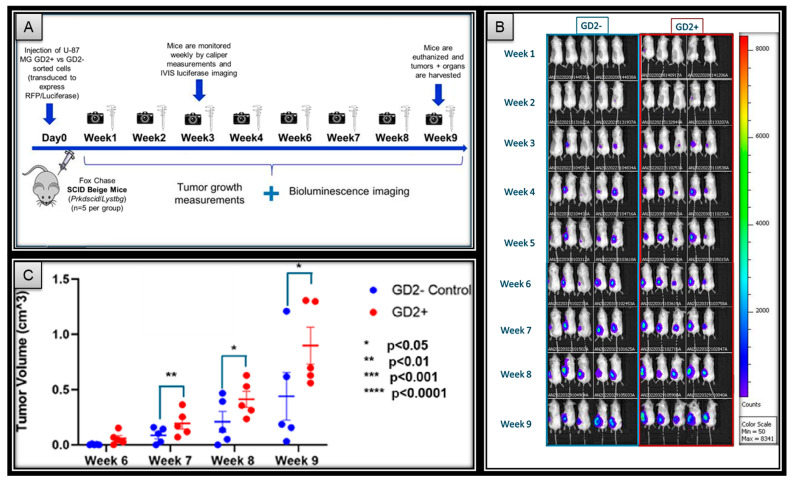
(**A**) In-vivo experimental design. Immunodeficient mice are implanted with tumor cells on day 0. Tumor growth is monitored weekly with caliper measurements and bioluminescence imaging. (**B**) In-vivo imaging demonstrates GD2 positivity is associated with earlier tumorigenesis. Clear signal is seen in all 5 GD2+ mice by week 3 compared to later weeks for GD2− mice. (**C**) GD2 positivity is associated with faster tumor growth, by caliper measurements, with statistical difference observed at weeks 7, 8, and 9.

To determine the effects of GD2 expression on the in vivo tumorigenesis potential in GBM, GD2-positive and -negative sorted RFP/luciferase transduced U-87 MG cells were implanted into the flanks of immunodeficient SCID–beige mice. The mice were then routinely monitored for tumor progression with weekly IVIS (In Vivo Imaging System) fluorescence imaging and caliper measurements (Figure 3A).

The IVIS imaging demonstrates a different rate of tumor development between GD2+ and GD2− cells. At week 3, all five mice in the GD2+ group had robustly positive luciferase tumor signals, compared to only three in the GD2− group. However, all five mice in the GD2− group eventually developed detectable signals by week 5 (Figure 3B). Although tumor palpation was performed on a weekly basis, caliper measurements were not possible until week 6 due to insufficient tumor mass. At the week 6 timepoint, all five mice in the GD2+ group and only two out of five mice in the GD2− group had palpable and measurable tumors. Furthermore, there was a statistically significant increase in the tumor volumes of the GD2+ group compared to the GD2− controls at weeks 6, 7, 8, and 9 (Figure 3C). The experiment was concluded on week 9, and all mice were euthanized per the IACUC-approved protocol.

### 2.3. Glioma Cells Exhibit GD2 Expression Plasticity

Previous experiments have shown that GD+ sorted glioma cells exhibit enhanced spheroid and tumor formation capabilities compared to GD2− sorted cells. However, the GD2− sorted group still retained the ability to form spheroids and tumors. This raises the questions as to whether GD2 expression could be spontaneously induced in GD2− cells and how malleable GD2 expression is in glioma. GD2 has been previously reported to vary with culture confluency and metabolic conditions in triple-negative breast cancer [12]. This variation with culture conditions is also seen in glioma cell lines with our typical U-87 MG GD2 populations, ranging from approximately 10% to 30% prior to experimentation (Supplementary Figure S2).

**Figure 4:**
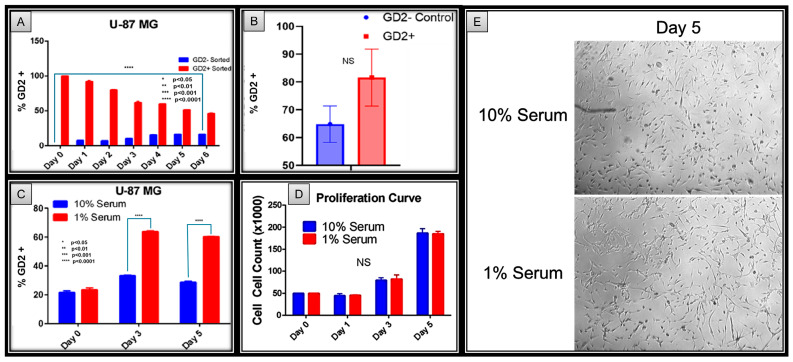
(**A**) GD2− cells can spontaneously generate GD2+ cells and vice versa. This demonstrates the plasticity of GD2 expression and provides an explanation to the phenomena observed in Figure 1C. (**B**) Endpoint in-vivo tumor analysis show high levels of GD2 expression and no statistical significance between GD2 expression of the two groups. (**C**) Low serum media increases GD2+ sub-population compared to complete media, suggesting a metabolic component for GD2 positivity. (**D**) Low serum media does not affect proliferation despite inducing GD2 positivity. (**E**) In addition to not affecting cellular proliferation rate, low serum media does not grossly affect cellular morphology as cells in both groups demonstrate similar characteristics such as their spindled shape and overall confluence.

We performed an initial cell culture experiment using GD2+ and GD2− flow cytometry sorted glioma cells that were seeded with either GD2− or GD2+ cells. The results revealed significant cellular plasticity under the following conditions:

1. When they were seeded with 100% GD2− cells: GD2 expression appeared by day 1, with the percentage of GD2+ cells increasing over time in culture.
2. When they were seeded with 100% GD2+ cells: Loss of GD2 expression was seen by day 1, with the percentage of GD2− cells increasing over time in culture (Figure 4A and Supplementary Figure S4A).

Furthermore, tumors from the cell-sorted in vivo experiments described in the previous section were analyzed for GD2 expression at endpoint. Like the in vitro experiments, tumors from GD2− seeded mice had a significant population of GD2+ cells (Figure 4B and Supplementary Figure S4B). Furthermore, qPCR analysis of endpoint tumors shows no statistically significant difference in the stem cell genes SOX2 and Oct4 (Supplementary Figure S5). These results suggest that glioma cells have the ability to undergo a plastic adjustment of GD2 expressivity regardless of the initial GD2 expression status.

GD2 expression in TNBC has been previously reported to be regulated by metabolic pathways and nutrient availability [12]. For scientific rigor, glioma cells were cultured in complete media (supplemented with 10% serum) or nutrient-deficient media (supplemented with 1% serum). Similar to previous reports, cells cultured in nutrient-deficient media had a significant increase in the GD2+ population compared to controls (Figure 4C and Supplementary Figure S4C). Furthermore, serum supplementation had a negligible effect on cellular proliferation (Figure 4D,E).

### 2.4. Natural Compounds Tuberosin, Broussonin B, and Glyceollin Have Anti-Spheroid Effects on Glioma Cells

The primary method for targeting GD2 utilizes immunotherapeutic techniques, including monoclonal antibodies, CAR-T cell therapy, and antitumor vaccines [10,16,17,18]. While triptolide, derived from the *Tripterygium* plant genus, has been demonstrated to inhibit GD3S, a key enzyme for GD2 synthesis, there remains a lack of molecular inhibitors specific for targeting GD2 [19,20]. To identify potential compounds for inhibiting cancer stem cell function, and thus potential GD2 inhibitors, a panel of natural compounds developed in collaboration with the USDA was used to screen for their effect on spheroid formation in glioblastoma cell lines.

**Figure 5:**
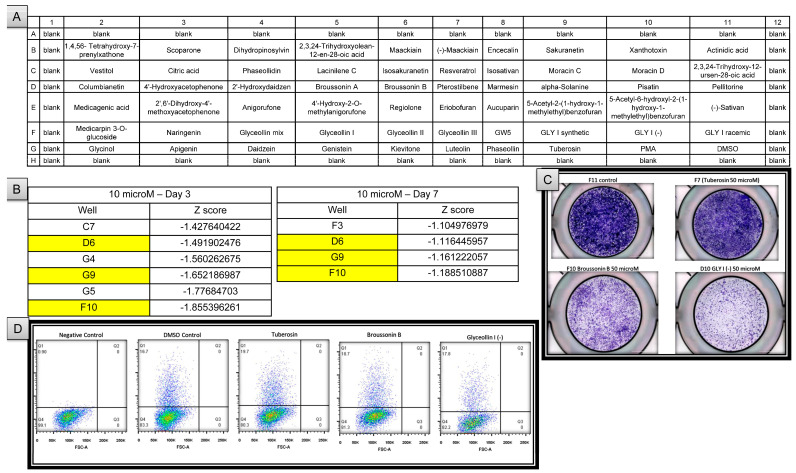
(**A**) USDA natural compounds. The table represents how the compounds were delivered in a 96-well plate dissolved with DMSO. (**B**) Phytochemicals identified to have largest anti-spheroid effect on glioblastoma cell lines. Highlighted compounds in wells D6, G9, and F10 had strong effects on spheroid formation on day 3 and day 7. (**C**) Lead compounds and their effect on 2D culture proliferation and morphology. (**D**) Lead compounds have minimal effect on GD2 expression at 10 µM after 72 h of treatment.

An initial screen for spheroid formation was performed using a panel of natural compounds developed in collaboration with the USDA on U-87 MG cells seeded into non-adherent, round-bottom 96-well plates (Figure 5A). Plates were then imaged on days 3 and 7 after treatment and analyzed for size. Spheroid measurements were obtained by ImageJ analysis, and standard (Z) scores were calculated. Cells treated with broussonin B, tuberosin, and glyceollin I were identified to have formed smaller spheres compared to controls on both days 3 and 7 after treatment (Figure 5B and Supplementary Figure S6).

To determine whether the effect on the spheroid size was due to cellular proliferation, U-87 MG cells were treated with the leading compounds in adherent culture conditions and imaged on day 7. Treatment with tuberosin showed minimal differences in the cellular confluency compared to the control group, suggesting a minimal effect on cell proliferation. In contrast, the treatment groups with broussonin B and glyceollin I exhibited visibly decreased confluency, indicating a potential decrease in cellular proliferation or an increase in cell death (Figure 5C).

Lastly, flow cytometry was performed on U-87 MG cells treated with lead compounds for 3 days in adherent culture conditions. Despite reducing spheroid formation capabilities, neither tuberosin, broussonin B, nor glyceollin I had an effect on the GD2 levels compared to the DMSO control group (Figure 5D). Altogether, this dataset suggests that tuberosin, broussonin B, and glyceollin may have an inhibitory effect on the function of cancer stem cells in glioblastoma but not directly on GD2 expression itself.

## 3. Discussion

GD2 has been reported to be a cancer stem cell marker for triple-negative breast cancer and has been identified to play significant roles in tumor growth and progression [21,22,23]. While GD2 is highly expressed in glioblastoma (GBM), its role as a cancer stem cell marker in this disease context remains controversial. There is an argument against GD2′s role as a GBM CSC marker in a prior report identifying no difference in the spheroid formation between GD2+ and GD2− sorted U-87 MG cells after 2 weeks of culture [11]. Upon repeating these initial experiments, we found that GD2, along with several well-known cancer stem cell markers, is highly expressed in the GBM cell lines U-87 MG and U-118 MG. Furthermore, our multi-timepoint experiments found no differences in the spheroid formation between GD2− and GD2+ GBM cells after 48 h of culture, consistent with earlier reports that showed no significant differences after 2–3 weeks. However, there was a decrease in the spheroid formation size in GD2− compared to GD2+ sorted cells at the 24 h timepoint. These results suggest that GD2+ glioma cells exhibit enhanced tumorsphere formation compared to GD2− cells, indicating a potential short-term difference that warrants further exploration.

Cancer stem cells have also been demonstrated to have increased motility in various culture environments [24,25]. Furthermore, it has been postulated that cancer stem cells are responsible, at least in part, for clinical metastasis due to an enrichment of motility pathways, such as FAK and mTOR signaling [26,27,28]. Our qPCR results demonstrate that for GD3S, the rate-limiting enzyme for GD2, the mRNA expression is correlated with known stem cell markers. Of note, CD133 and nestin are positively correlated with GD3S expression in both cell lines. To expand upon the initial spheroid results, transwell migration assays were performed to determine differences in the motility between GD2+ and GD2− GBM cells. The results demonstrated an increase in the total cell migration of GD2+ sorted compared to GD2− sorted cells. Altogether, the in vitro results are consistent with findings for other cancer types, where GD2 expression has been associated with increased tumorigenesis and migratory capabilities [29,30,31].

In addition to in vitro spheroid formation and cellular motility, CSCs have also been characterized as having an increased in vivo tumorigenic potential [32,33,34]. To expand upon our in vitro results, we implanted mice with GD2+ glioma cells to determine the effects of GD2 expression on tumor formation. IVIS luminescence imaging determined faster tumor initiation rates, with signals detected in all five GD2+ mice, compared to three GD2− mice, by week 3. Furthermore, caliper measurements demonstrated that the GD2+ group also grew larger tumors at faster rates compared to the GD2− group. Overall, the in vivo results are aligned with the existing literature as well as our in vitro data, supporting the hypothesis that GD2 is a cancer stem cell marker for GBM [12,21,29,35,36].

So far, our in vitro and in vivo experimental results demonstrate the increased capability for the spheroid formation, migration, and tumorigenesis of GD2+ GBM cells. However, GD2− cells were still able to form spheroids, migrate, and grow tumors, although not as effectively as GD2+ sorted cells. This suggests that while GD2 enhances tumor-initiating and migration capabilities, GD2− cells could potentially perform the functions of cancer stem cells. The existing literature explains this phenomenon, with reports demonstrating that GD2− cells can spontaneously acquire GD2 expression over time. One notable report demonstrates that environmental factors, such as nutrient availability, appear to influence GD2 expression in TNBC [12]. Our experimental results identified the ability of GD2− cells to generate GD2+ cells in culture, and vice versa, aligning with the existing literature. Furthermore, low-serum culture conditions also enhanced GD2 positivity, with no discernable effect on the cellular proliferation, survival, or gross morphology.

Given the functional implications of cancer stem cells in glioma, targeting cancer stem cells and potentially GD2-expressing cells represents a promising therapeutic avenue. Current strategies predominantly focus on GD2-directed immunotherapies, including monoclonal antibodies and CAR-T cell therapies. However, small-molecule inhibitors of GD2 remain scarce [20,37]. Our natural compound screening identified three potential inhibitors—tuberosin, broussonin B, and glyceollin I—that significantly reduced glioblastoma spheroid formation. Interestingly, while broussonin B and glyceollin I may exert antitumor effects by decreasing proliferation, tuberosin reduced spheroid formation without affecting cell confluency, suggesting a unique mechanism of action. However, none of the compounds altered GD2 expression levels, implying that their effects are independent of GD2 downregulation. Further studies are needed to elucidate their mechanisms and potential for clinical translation.

One limitation of our study is the use of cell lines. While the original study that inspired this manuscript used the patient-derived GBM cell lines 464T and 532T, we utilized U-87 MG and U-118 MG [11]. Although these cell lines are widely used as models in the research community, we must recognize that they were originally derived decades ago and have undoubtedly undergone extensive passaging, drift, and loss of tumor heterogeneity. To address these concerns, we confirmed that both U-87 MG and U-118 MG still maintained the capacity to generate GD2− and GD2+ subpopulations by flow cytometry. Furthermore, qPCR analysis demonstrated the increased stem cell marker expression of GD2+ sorted cells. Lastly, experiments in this manuscript were performed within five passages with strict cell culture conditions to minimize variability and phenotypic drift.

Future studies should further build on these findings by validating the role of GD2 through inhibition or knock-down/knock-out models. Furthermore, patient-derived xenograft and organoid models that better capture the heterogeneity and microenvironmental influences of glioblastoma would also strengthen GD2′s role as a cancer stem cell marker. Although NFkB and FAK signaling have been implicated in GD2 regulation in triple-negative breast cancer, mechanistic studies are also warranted to define the signaling pathways downstream of GD2 that drive stemness and motility [27,29]. In parallel, further investigation of the natural compounds identified in our screen—particularly tuberosin, which reduced spheroid formation independent of changes in GD2 expression—will be essential to clarify their mechanisms of action and assess their efficacy in vivo. Additionally, determining the multipotency of GD2 and how these compounds may affect it would also be an interesting avenue to pursue. Finally, understanding the plasticity between GD2^+^ and GD2^−^ populations, and the environmental factors that regulate this dynamic, will be critical for the development of durable GD2-targeted therapies.

## 4. Materials and Methods

### 4.1. Cell Culture and Serum Deprivation Assay

The human glioblastoma cell lines U-87 MG (obtained from collaborator Dr. Jane Cavanaugh at Duquesne University, Pittsburgh, PA, USA) and U-118 MG (obtained from collaborator Dr. Reza Izadapanah at Tulane University, New Orleans, LA, USA) were propagated in a complete cell culture medium comprising Dulbecco’s Modified Eagle Medium (DMEM) (Corning 10-013-CV, Corning, NY, USA) and 10% (50 mL) Cosmic Calf Serum (CCS) (HyClone SH30087.03) (ThermoFisher Scientific, Waltham, MA, USA). Experiments were performed within 5 passages of thawing, and cells were routinely checked for *mycoplasma* using the Microsart^®^ RESEARCH Mycoplasma real-time PCR kit (Sartorius SMB95-1005, Göttingen, Germany) prior to initiation of experiments and routinely at ~6 month intervals by the Burow Lab group at Tulane University School of Medicine. Cells were cultured using a standard technique: incubation at 37 °C at 5% CO_2_, passaged at or prior to 80% confluency using trypsin (Corning 25053CI, Corning, NY, USA), and handled with a sterile technique.

Alternatively, serum deprivation assays were performed with DMEM containing 1% (5 mL) CCS. Growth curves were generated by hemocytometer, with brightfield images generated by the Nikon Eclipse microscope (Nikon, Tokyo, Japan).

U-87 MG cell lines utilized for animal experiments were transduced to express RFP and luciferase with lentivirus (GenTarget LVP324, San Diego, CA, USA) and underwent puromycin selection, 1 µg/mL for 48 h, prior to experiments. RFP/luciferase cells are similar to parental cell lines in the behaviors and functions examined in this manuscript. Experiments were performed in triplicates.

### 4.2. Flow Cytometry Analysis and Fluorescence-Activated Cell Sorting (FACS)

To summarize, cells (~1 × 10^6^) were incubated with allophycocyanin (APC)-conjugated anti-GD2 antibody (BioLegend 357306, San Diego, CA, USA) for 30 min on ice in the dark after trypsinization and washing with PBS (Corning 21-040, Corning, NY, USA). Cells were then washed with ~1 mL of PBS containing 4′,6-diamino-2-phenylindole (DAPI; 1 μg/mL) and resuspended in ~300 µL of PBS or another FACS-approved buffer. For sorting large samples, the numbers of cells and reagents were appropriately scaled upwards. Flow cytometry analysis and cell sorting of U-87 MG and U-118 MG were performed using the BD Fortessa LSRII and BD FACSAria II Cell Sorter, respectively (BD, Franklin Lakes, NJ, USA). Gating analysis and images for figures were generated using FlowJo software (version 11, BD, Franklin Lakes, NJ, USA).

### 4.3. Spheroid Assay

GD2− or GD2+ sorted U-87 MG cells were plated at 10 × 10^3^ in 0.5 mL of complete media, as described above, into 24-well ultra-low adherent plates (Costar 3473, Corning, NY, USA) and imaged at 24 h intervals after seeding using the Zeiss cell imaging system (Jena, Germany). For screening assays, 2 × 10^3^ cells in 100 µL of media were seeded into 96-well ultra-low adherent plates (S-bio MS9096, Hudson, NH, USA). Treatment was initiated 24 h after seeding. Spheroid analysis was performed using ImageJ software to measure the areas of images (version 1.52s, NIH, Bethesda, MD, USA). Briefly, ImageJ was used to identify the area of a spheroid image from a microscope, and the number of pixels in the spheroid was counted. Standard (Z) scores were then calculated using Excel.

For the screening assays, all natural compounds were tested at 10 µM concentrations with DMSO as the vehicle. An equal volume of DMSO was used for the treatment control groups. The treatment groups were tested in quadruplicates.

### 4.4. Real-Time Reverse Transcription Polymerase Chain Reaction (RT-PCR)

A total of 1 × 10^6^ sorted GD2− and GD2+ U-87 MG or U-118 glioma cancer cells were processed for RNA extraction and cDNA generation using the Zymo Quick-RNA Miniprep kit (R1055, Irvine, CA, USA) and Quantabio qScript cDNA Synthesis kit (95047-100, Beverly, MA, USA). Real-time RT-PCR was performed using PerfeCTa SYBR Green SuperMix (95054-500, Beverly, MA, USA). Technical and biology triplicates were used. Primer sequences are listed below (Table 1).

### 4.5. Crystal Violet Staining

An amount of 2 × 10^3^ in 100 µL was seeded into 96-well plates (Corning 3595, Corning, NY, USA). After 24 h of incubation, compound or DMSO control in 100 µL of media was added to their respective wells. After 7 additional days of incubation, the cells were fixed with 10 µL of 25% glutaraldehyde solution (Fisher Scientific 02957-1, Pittsburgh, PN, USA) at room temperature for 15 min, washed with water, stained with 50 µL of diluted 0.1% crystal violet solution (Sigma V5265, St. Louis, MO, USA) for 1 h, washed with water 3X, and allowed to air dry. Plates were then imaged using the BioTek Cytation 5 system (Agilent Technologies, Santa Clara, CA, USA). Triplicates were used.

### 4.6. Migration Assay

Migration assays were performed using 24-well plates (Corning 3512, Corning, NY, USA) with 3 µm pore membrane inserts (Corning 3402, Corning, NY, USA). Briefly, GD2− and GD2+ sorted U-87 MG or U-118 MG cells in DMEM containing 1% CCS were seeded (1 × 10^4^ per chamber) into the upper chambers with 10% CCS supplemented media in the lower chambers. After incubating for 16 h at 37 °C in a 5% CO_2_ incubator, each membrane was carefully removed from the insert, fixed with glutaraldehyde solution (Fisher Scientific 02957-1, Pittsburgh, PN, USA) and stained with crystal violet solution (Sigma V5265, St. Louis, MO, USA), as described in Section 4.5. Images were captured using the Zeiss cell imaging system, and migrated cells were hand-counted. Triplicates were used.

### 4.7. Graphs and Statistics

Graphs and statistics were generated by inputting data values into GraphPad Prism software (version 9.0.0, Boston, MA, USA) unless otherwise stated. Briefly, raw measurements were entered into GraphPad Prism software. Means, standard deviations, and standard errors were automatically calculated. Paired *t*-test analysis was performed to calculate *p*-values for differences between two groups, and an ANOVA was used for calculating the *p*-values between two groups with different timepoints, with *p* < 0.05 being the cutoff for statistical significance.

### 4.8. In Vivo Tumor Growth

All experiments involving animals were approved by and conducted in accordance with the policies of the Institutional Animal Care and Use Committee (IACUC) of Tulane University School of Medicine under Protocol ID 1515 (approved on 18 April 2022; Form ID 4449). Sorted RFP/luciferase-expressing U-87 MG, GD2+ vs. GD2− cells were resuspended in Matrigel Matrix (Corning 354277, Corning, NY, USA). An amount of 2 × 10^6^ cells per 100 µL of Matrigel was then injected into the flanks of Fox Chase SCID–beige mice (Charles River CB17.Cg-*Prkdc^scid^Lyst^bg-J^*/Crl) (n = 10; 5 mice per group) (all female at 10 weeks of age). The initial tumor injection was performed with groups blinded to the injector. Tumor measurements were performed weekly using calipers, with the tumor volume being approximated by $V=\frac{4}{3}\pi {\left(average\;radius\right)}^{3}$, where $average\;radius=\;\frac{length+width+depth}{6}$. Mice were also injected with 100 µL of PBS containing luciferin (Sigma-Aldrich L6882) and imaged for bioluminescence using the IVIS. All mice were euthanized, with the experiment ended after the first mouse reached a tumor burden >4 cm in diameter or >20% of the mouse mass (9 weeks post-implant).

Harvested tumors were digested mechanically via chopping with sterile scalpel blades (Fine Science Tools 10021-00, Foster City, CA, USA) and enzymatically via dispase (Stemcell Technologies 07923, Vancouver, BC, Canada) and collagenase (Sigma C9891, Burlington, MA, USA). Red blood cell lysis was performed with Red Blood Cell Lysis Solution (Miltenyi Biotec 130-094-183, Bergisch Gladbach, Germany). To briefly summarize, tumors were mechanically digested and were then added to 10 mL/gram of enzyme solution and incubated for 3 h with 175 rpm shaking at 37 °C. The product was then sieved through a 70 µm filter, washed with PBS, and pelleted. RBC lysis solution was then added, then a PBS wash, and then it was pelleted for a final time. The resulting pellet was then used for subsequent analyses.

## 5. Conclusions

In conclusion, our study highlights the role of GD2 as a cancer stem cell marker in glioblastoma, demonstrating its correlation to tumorsphere formation, migration, and tumorigenicity. Furthermore, the observed plasticity of GD2 expression suggests that any therapeutic strategies developed for targeting cancer stem cells must account for GD2′s phenomenon of being dynamically expressed. Additionally, our identification of natural compounds with anti-spheroid effects opens new avenues for glioblastoma cancer stem cell targeting. Future work should focus on delineating the molecular pathways linking GD2 to glioblastoma progression and exploring how the mechanisms by which the natural compounds identified inhibit GD2 cancer stem cell functions.

## Figures and Tables

**Table 1:** qPCR primer sequences.

Gene	Forward	Reverse
*ß-Actin*	CACCATTGGCAATGAGCGGTTC	AGGTCTTTGCGGATGTCCACGT
*CD44*	CCAGAAGGAACAGTGGTTTGGC	ACTGTCCTCTGGGCTTGGTGTT
*CD133*	AGAGCTTGCACCAACAAAGTACAC	AAGCACAGAGGGTCATTGAGAGA
*GD3S*	GGAAGAGCATGTGGTATGACGG	CAGAATCCCACCATTTCCCACC
*NANOG*	CTCCAACATCCTGAACCTCAGC	CGTCACACCATTGCTATTCTTCG
*Nestin*	TCAAGATGTCCCTCAGCCTGGA	AAGCTGAGGGAAGTCTTGGAGC
*OCT4*	CAAAGCAGAAACCCTCGTGC	AACCACACRCGGACCACATC
*SOX2*	GCTACAGCATGATGCAGGACCA	TCTGCGAGCTGGTCATGGAGTT

## Data Availability

All research data was generated by our group at Tulane University and is available upon request.

## Supplementary Materials

The following supporting information can be downloaded at: https://www.mdpi.com/article/10.3390/ijms27083490/s1.
