# Supplementary material for: GD2 Identifies Cancer Stemness in Glioblastoma and Phytoalexin Library Screen Identifies Potential Novel Natural Inhibitors
Source: Int J Mol Sci. 2026 Apr 14;27(8):3490. doi: 10.3390/ijms27083490 (PMC13115847; doi:10.3390/ijms27083490)
Supplement: Supplementary file 1 [file ijms-27-03490-s001.zip › ijms-4205996-supplementary.pdf]

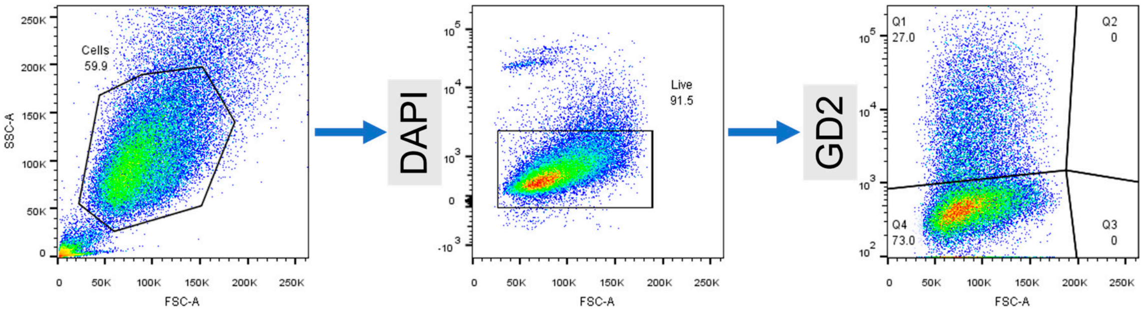

Supplementary Figure S1: Gating strategy for GD2- vs GD2+ flow assisted cell sorting. GD2 antibody is conjugated with APC.

Tube: SORT

Population	#Events	%Parent	%Total
All Events	53,697	####	100.0
SINGLET	36,321	67.6	67.6
SCATTER	34,312	94.5	63.9
DAPI neg	29,842	87.0	55.6
GD2+	7,145	23.9	13.3
GD2-	8,828	29.6	16.4

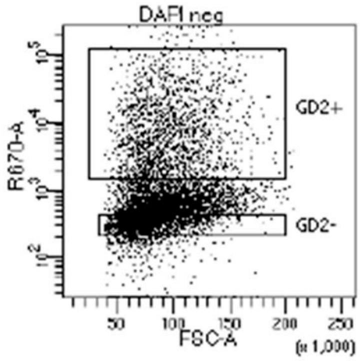

Tube: SORT

Population	#Events	%Parent	%Total
All Events	50,000	####	100.0
SINGLET	34,907	69.8	69.8
SCATTER	32,167	92.2	64.3
DAPI neg	31,681	98.5	63.4
GD2+	5,218	16.5	10.4
GD2-	5,312	16.8	10.6

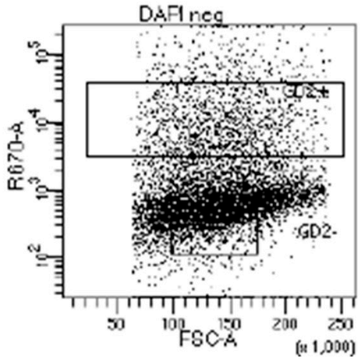

Supplementary Figure S2: GD2 expression can vary. These figures show analysis at two different time points with one being 24% GD2+ and the other being 16.5% GD2+.

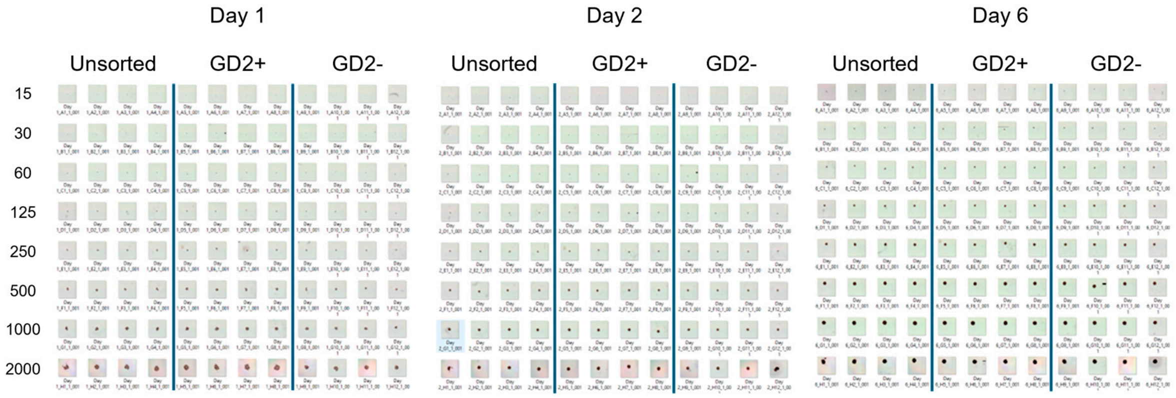

Supplementary Figure S3: Variable seeding of GD2 sorted U-87 MG cells and spheroid formation. The y-axis labels represent number of cells seeded.

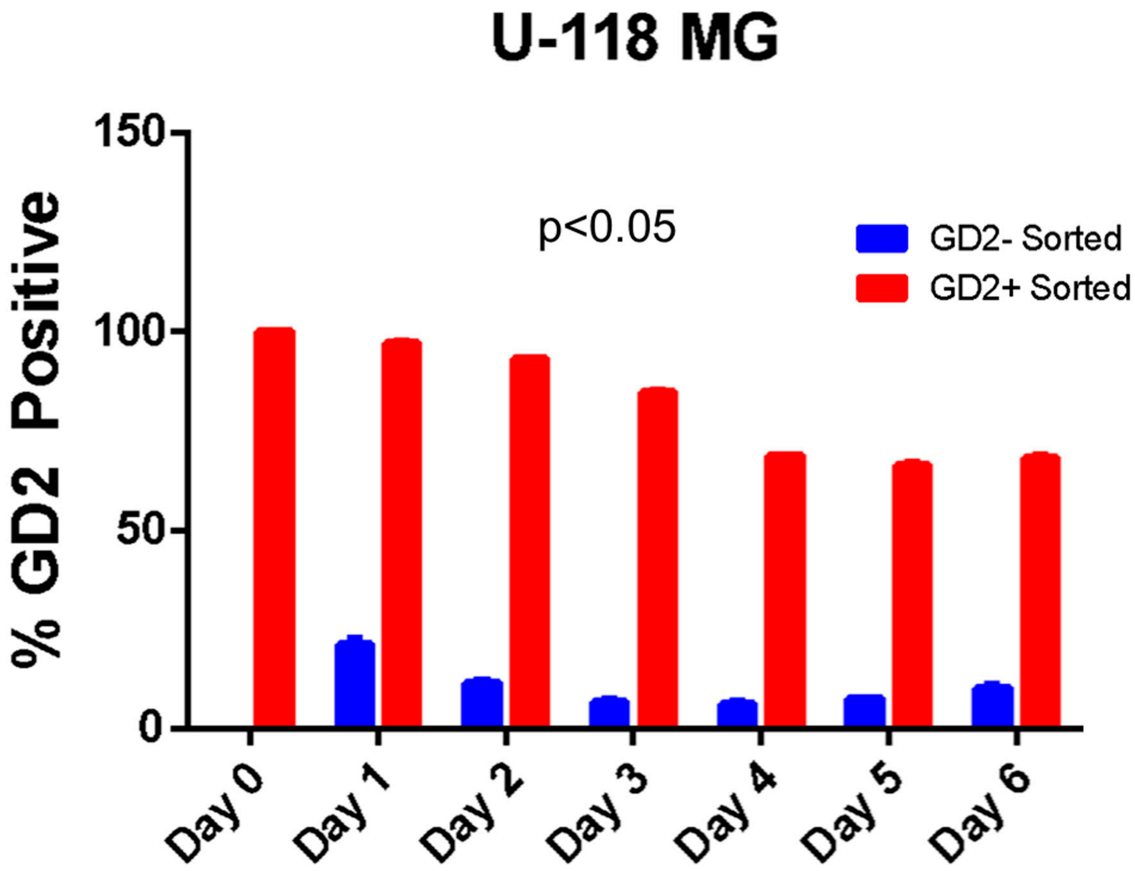

Supplementary Figure S4A: U-118 MG GD2- sorted cells can spontaneously generate GD2.

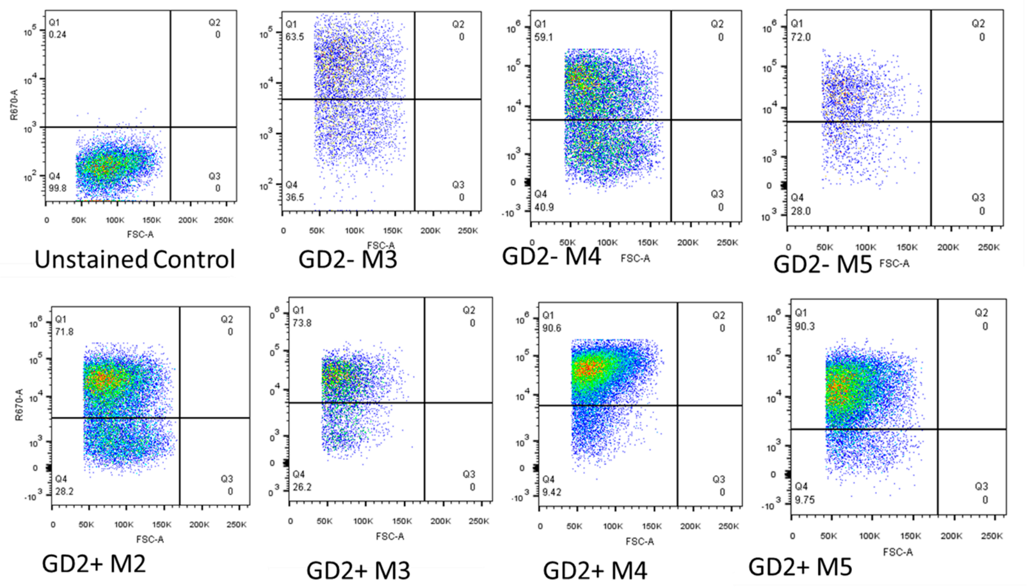

Supplementary Figure S4B: Flow cytometry analysis of GD2 in U-87 MG endpoint tumor analysis.

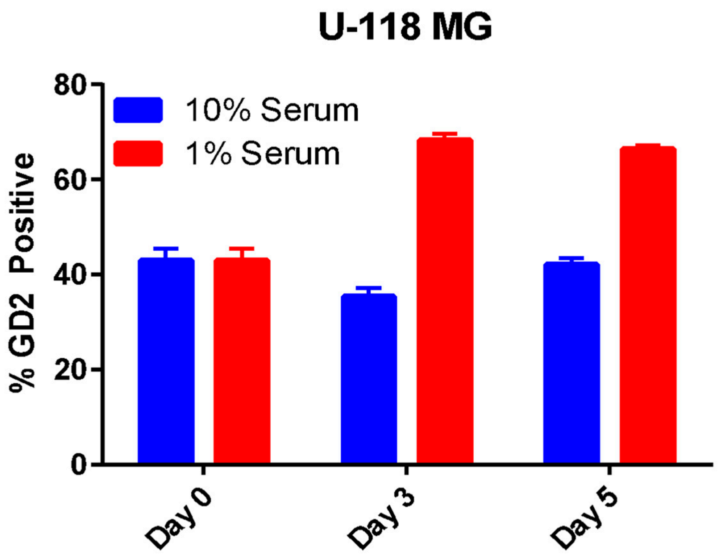

Supplementary Figure S4C: Serum deficient media induces GD2 expression in U-118 MG.

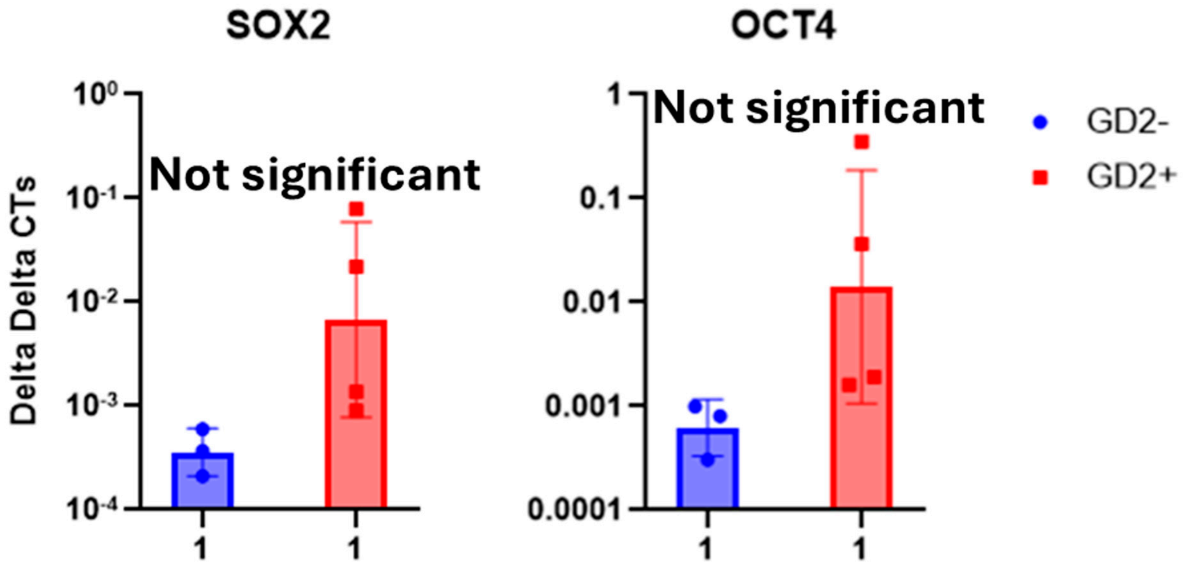

Supplementary Figure S5: Endpoint tumor analysis show no significant difference in cancer stem cell gene expression.

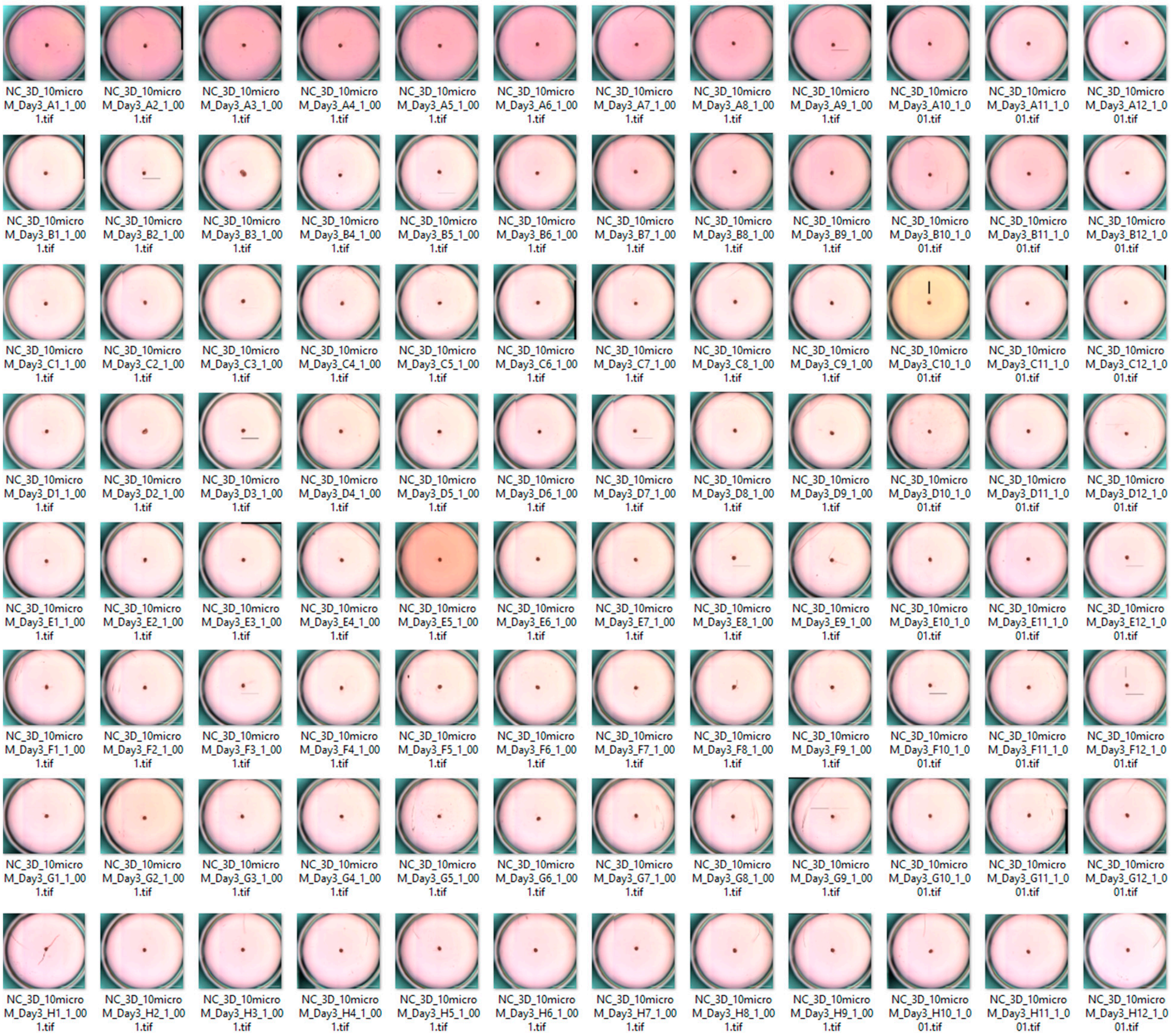

Supplementary Figure S6: Scanned plate of 72-hour, 10μM treated U87-MG neurospheres.
